# Age-related epidemiology and outcomes of sepsis in Japanese critical care units: a nationwide administrative claims database study

**DOI:** 10.1186/s40560-025-00837-4

**Published:** 2025-12-17

**Authors:** Hirotada Kobayashi, Mayuko Tonai, Toshiyuki Karumai, Atsushi Shiraishi, Kiyohide Fushimi, Yoshiro Hayashi

**Affiliations:** 1 Department of Intensive Care Medicine, Kameda Medical Center, 929 Higashi-cho, Kamogawa, Chiba 296-8602 Japan; 2 Department of Clinical Epidemiology and Health Economics, School of Public Health, The University of Tokyo, Tokyo, Japan; 3 Emergency and Trauma Center, Kameda Medical Center, Kamogawa, Japan; 4 Department of Health Policy and Informatics, Graduate School of Medical and Dental Sciences, Institute of Science Tokyo, Tokyo, Japan

**Keywords:** Administrative data, Ageing population, Epidemiology, Critical care resource, Sepsis

## Abstract

**Background:**

The global rise in the elderly population presents unique challenges in intensive care, including treatment decisions and end-of-life care due to physiological, ethical, and social complexities. However, data on the very elderly remain limited, highlighting the need for real-world, large-scale studies. We aimed to characterize the age-related epidemiology and outcomes of sepsis in Japan using a nationwide administrative claims database.

**Method:**

We conducted a retrospective cohort study using the Japanese Diagnosis Procedure Combination database for fiscal years 2010–2019. Sepsis was defined by infection-related ICD-10 codes, blood culture testing, and administration of antimicrobials within a three-day window around admission to critical care unit. Patients were stratified into 10-year age groups, and logistic regression was used to assess the association between age and in-hospital mortality.

**Results:**

Among 1,880,275 critical care patients with infection-related diagnosis, 511,848 met the sepsis criteria. The median age was 76 years, and 40.0% were aged ≥ 80 years. ICU and hospital mortality increased with age, reaching 9.5% and 24.9%, respectively, in patients aged ≥ 90 years. Compared to patients aged ≤ 29 years, those aged ≥ 90 had an adjusted odds ratio of 4.75 (95% CI 4.37–5.16) for in-hospital mortality. The proportion of patients discharged home declined with age, falling to 29.4% in the ≥ 90 group. Use of organ support, including vasopressors and mechanical ventilation, was inversely related to age.

**Conclusions:**

Sepsis in Japanese critical care units demonstrated substantial age-related differences in epidemiology, treatment, resource utilization, and outcomes. The disproportionately high mortality and reduced treatment intensity among the oldest patients underscore the complex clinical and ethical considerations involved in managing sepsis in aging societies. These findings emphasize the urgent need to adapt sepsis care models and critical care resource planning for the rapidly aging population in Japan and similar nations.

**Supplementary Information:**

The online version contains supplementary material available at 10.1186/s40560-025-00837-4.

## Background

The global population is aging at an unprecedented rate, leading to a growing number of elderly individuals requiring critical care. This demographic shift has resulted in a substantial rise in the proportion of the “oldest old” (≥ 85 years) admitted to intensive care units (ICUs), posing distinct challenges in critical care practice. These challenges are particularly evident in resource allocation, treatment decision-making, and end-of-life care, where clear guidelines are often lacking [1–5]. The underlying causes are multifactorial, including physiological vulnerabilities, a higher burden of comorbidities, ethical complexities in end-of-life decision-making, and broader social determinants of health [6–13].

Numerous epidemiological studies have examined the outcomes associated with sepsis. In particular, research on critically ill elderly patients with sepsis has identified advancing age as a key predictor of adverse outcomes [4, 8, 9, 12, 14]. However, the epidemiology of sepsis in the very elderly population remains poorly characterized due to their underrepresentation in clinical trials and the heterogeneity of prior study populations [15, 16]. Furthermore, few studies have investigated this age group in the context of real-world intensive care practice at a national scale. As the global population continues to age, a nuanced understanding of sepsis outcomes across the full age spectrum including the oldest patients is essential to guide clinical decision-making and healthcare planning.

Japan, with the highest proportion of elderly individuals in the world, provides a unique and timely context for such an investigation. We hypothesized a nationwide description of sepsis across age groups in Japan would provide understanding age-related differences in epidemiology, treatment practice, resource utilization, and outcomes. This study aimed to investigate age-related epidemiology and patients’ outcomes following sepsis in Japanese critical care units and to offer critical insight into the burden and management of sepsis across age groups in a super-aged society, using a large, nationwide administrative claim database.

## Methods

### Study design and data source

This retrospective descriptive study was conducted using the Japanese Diagnosis Procedure Combination (DPC) database spanning the fiscal years 2010 to 2019 (from April 2010 to March 2020). The DPC database is a comprehensive national repository of hospital discharge records and administrative claims from Japanese hospitals [18]. As of 2023, it collects data from over 1700 hospitals (approximately 826,000 beds), covering 93% of all acute care hospitals in Japan. This extensive database includes information on diagnoses, daily medical interventions, administered medications, medical device usage, and discharge outcomes, all recorded using standardized codes. Diagnostic codes are classified according to the International Classification of Diseases, 10th Revision (ICD-10), and capture primary diagnoses, comorbidities, and other resource-intensive conditions.

### Patient population, inclusion criteria, and exclusion criteria

The interested population included adult patients with sepsis who were admitted to critical care units. Sepsis was defined as the presence of infection-related ICD-10 codes, accompanied by antibiotic administration, and blood culture testing within the three-day window spanning the day before, the day of, and the day after critical care unit admission, following a previously established approach [19]. The inclusion criteria were as follows

- Age ≥ 18 years
- At least one ICD-10 code for an infectious disease with the potential to cause sepsis during hospitalization, generated using the Delphi method based on agreements among three independent intensivists (HK, MT, and TK) [19].
- Admission to a critical care unit, including an intensive care unit (ICU), high dependency unit (HDU), or emergency ICU (EICU; a unit adjacent to the emergency department where emergency physicians provide ongoing critical care management).
- Blood culture testing and antimicrobial administration within the defined timeframe.

Patients with incomplete data on discharge outcomes were excluded from the analysis.

### Variables

Baseline variables included age, sex, height, weight, ambulance transport status, and Elixhauser comorbidity score, calculated using the van Walraven method from diagnostic codes [20–22]. Clinical characteristics included diagnoses, primary source of sepsis, organ support use, and surgical procedures under general anesthesia. Each hospitalization was assigned up to six diagnostic codes: primary diagnosis, diagnosis leading to admission, most and second most resource-intensive diagnoses, comorbidities at admission, and complications that developed post-admission. In particular, “diagnosis leading to admission” and “comorbidities at admission” reflected patients’ baseline clinical status.

### Primary and secondary endpoints

Therapeutic interventions such as vasopressors, mechanical ventilation, and renal replacement therapy (RRT) were recorded daily throughout hospitalization. Organ support use was summarized from the day of critical care unit admission until discharge. Our primary outcome was hospital mortality and secondary outcomes included 14-day critical care mortality, hospital length of stay, days of critical care billing (capped at 14 days for ICU/EICU stay under Japanese reimbursement system), organ support use in critical care units, days on organ support after critical care units’ admission, and discharge destination. The maximum critical care billing period was set at 14 days in accordance with Japan’s medical reimbursement system, which limits reimbursable ICU/EICU stay to 14 days (and HDU to 21 days including any ICU days,). The specific diagnosis and procedure codes used in this study are listed in Supplementary Appendix 1. To further explore resource utilization patterns by age, we described discharge outcomes stratified by the use of organ support therapies.

### Statistical analysis

Descriptive statistics were reported as counts with percentages or medians with interquartile ranges (IQRs), as appropriate. Patients were stratified into 10-year age groups to illustrate age-related patterns. To explore association between age and in-hospital mortality, we fitted mixed effect logistic regression models using the youngest adult patients (aged 18–29 years) as the reference group, assumed patients with least comorbidities and frailty.

Two adjusted models were developed:

- Model 1: Adjusted for sex and the Elixhauser comorbidity score (fixed effects), with hospital modeled as a random effect to account for clustering
- Model 2: Further adjusted for vasopressor use, mechanical ventilation, and RRT on the day of ICU/HDU/EICU admission as surrogate markers of disease severity.

Results were reported as odds ratio (OR) with 95% confidence intervals (CIs). Given the use of a large-scale nationwide administrative database, a formal sample size calculation was not deemed necessary. All analyses were approved by the authors prior and conducted as complete-case analysis using R, version 4.2.2 (R Development Core Team, Vienna, Austria, 2022).

## Results

### Patient characteristics

**Fig. 1:**
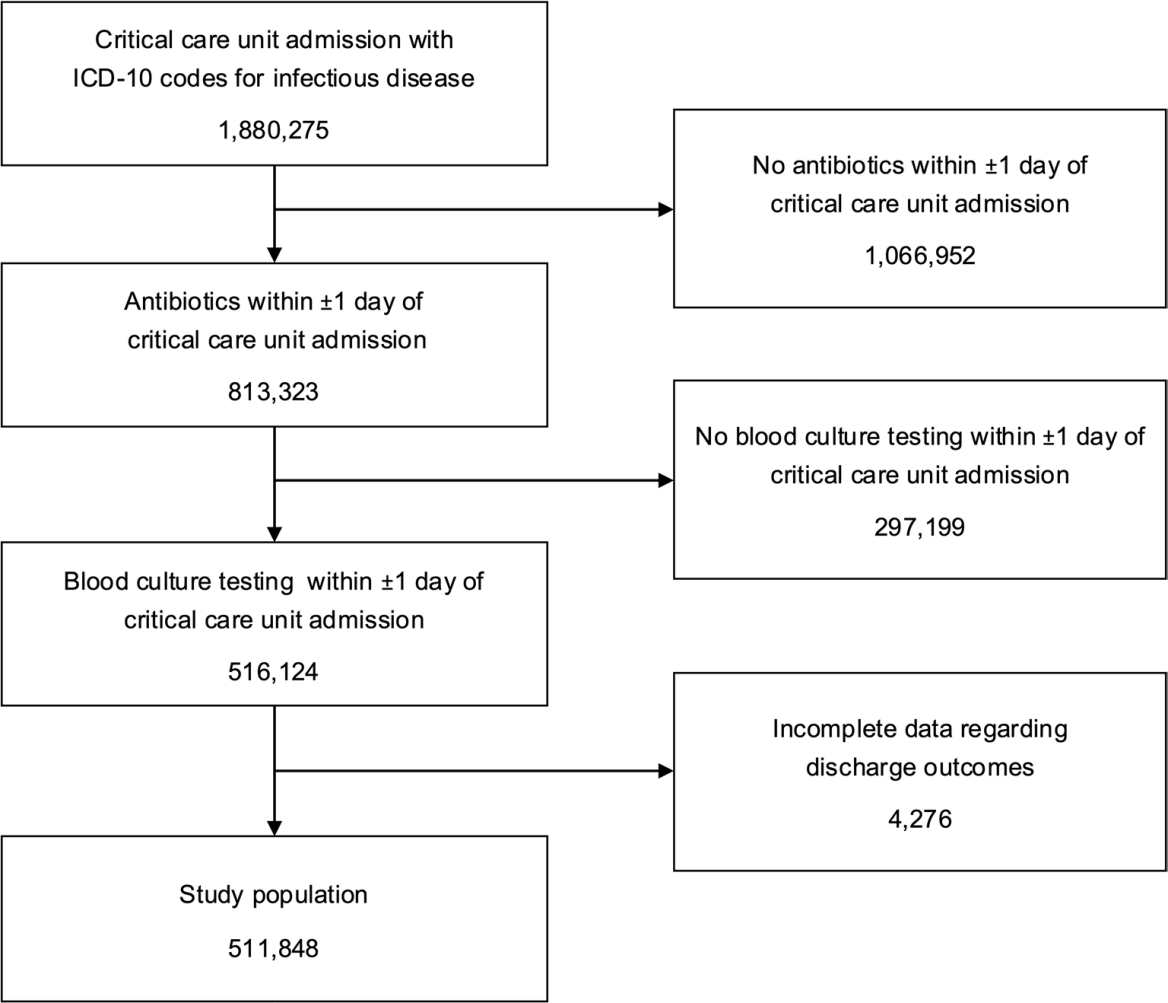
Patient flowchart. Flowchart shows patient selection and inclusion criteria. *ICU* intensive care unit, *HDU* high dependency unit, *EICU* emergency intensive care unit

During the study period, 1,880,275 patients were admitted to critical care units with ICD-10 codes indicating infectious diseases potentially leading to sepsis. Among them, 511,848 met the inclusion criteria, having undergone blood culture testing and received antimicrobial therapy within the three-day period surrounding their critical care unit admission (Fig. 1). The median age of the cohort was 76.0 years and 40.6% were female. Surgical patients accounted for 21.5% of the total population. Patients were treated in ICUs (31.5%), HDUs (18.2%), and EICUs (50.3%) (Table S1).

**Fig. 2:**
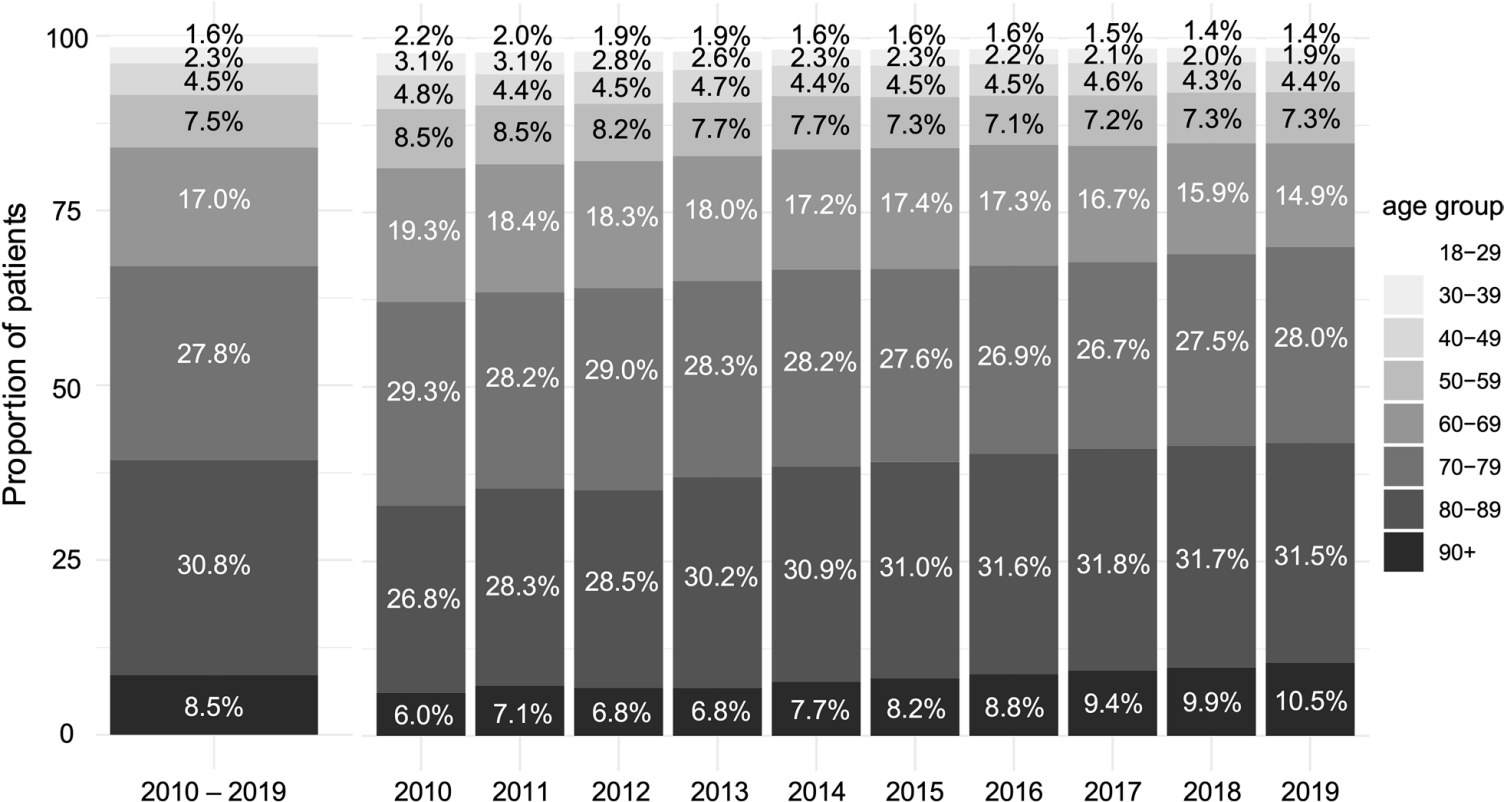
Age distribution of study patients. The left panel shows the age distribution of the entire cohort during the study period, whereas the right panel illustrates trends in age structure from fiscal year 2010 to 2019

The age distribution was skewed towards older adults, with 201,282 patients (40%) aged ≥ 80 years. Among them, 43,694 (8.5%) were aged ≥ 90 years, including 1171 (0.2%) aged ≥ 100 years. The largest age group was 80–89 years (30.8%), followed by 70–79 years (27.8%). Over the 10-year study period, the proportion of patients aged ≥ 70 years increased from 62.1% to 70.0% (Fig. 2). While females accounted for less than half of the patients in age groups up to 89 years, their proportion rose to 62.2% among patients aged ≥ 90 years.

Respiratory infections were the most common source of sepsis across all age groups, with prevalence ranging from 36.9% in the 40–49 age group to 51.2% in patients aged ≥ 90 years. Intra-abdominal infections were the second most common source, occurring in 20.8% of patients aged ≤ 29 years and 27.4% in those aged 60–69 years. In contrast, central nervous system (CNS) infections were more frequent in younger patients, decreasing from 20.4% in the youngest age group to just 2.0% in those aged ≥ 90 years. Urogenital infections, by contrast, increased with age, rising from 6.0% in the youngest age group to 17.7% among those ≥ 90 years.

Surgical procedures under general anesthesia were most frequently performed in the 40–49 age group, with 31.0% of patients in that group undergoing surgery. Additional details on patient characteristics by age group are presented in Table 1 and Table S2.

**Table 1:** Background of patients

	18–29 N = 83,22	30–39 N = 11,790	40–49 N = 23,033	50–59 N = 38,410	60–69 N = 86, 928	70–79 N = 142,083	80–89 N = 15, 7588	90 + N = 43,694
Female	3437 (41.3)	4780 (40.5)	8260 (35.9)	12,500 (32.5)	28,099 (32.3)	51,347 (36.1)	72,238 (45.8)	27,189 (62.2)
Age, years	24 [21, 27]	35 [33, 38]	45 [43, 47]	55 [53, 58]	65 [63, 68]	75 [72, 77]	84 [82, 86]	92 [91, 94]
Weight, kg	55 [44, 65]	60[49, 72]	62 [50, 75]	60 [50, 71]	56 [47, 66]	53 [44, 62]	48 [40, 57]	43 [36, 50]
BMI	20.8 [18, 24]	22 [19, 26]	23 [19, 27]	23 [20, 26]	22 [19, 25]	22 [19, 25]	21 [18, 24]	20 [18, 23]
Elixhauser score^a^	3 [0, 6]	3 [0, 6]	3 [0, 7]	4 [0, 8]	4 [0, 8]	4 [0, 8]	5 [0, 7]	5 [0, 7]
Surgical patients^b^	2205 (26.5)	3270 (27.7)	7151 (31.0)	11,558 (30.1)	23,744 (27.3)	31,850 (22.4)	25,844 (16.4)	4343 (9.9)
Hospital onset	974 (11.7)	1455 (12.3)	2819 (12.2)	4965 (12.9)	11,853 (13.6)	16,947 (11.9)	11,376 (7.2)	1440 (3.3)
Unit type								
ICU	2990 (35.4)	4500 (37.5)	8880 (38.2)	14,943 (38.5)	33,060 (37.7)	48,139 (33.6)	41,790 (26.3)	7779 (17.6)
HDU	1102 (13.0)	1521 (12.7)	3264 (14.0)	5646 (14.6)	13,732 (15.7)	24,919 (17.4)	32,117 (20.2)	11,442 (25.9)
EICU	4361 (51.6)	5971 (49.8)	11,117 (47.8)	18,174 (46.9)	40,802 (46.6)	70,033 (48.9)	84,946 (53.5)	24,896 (56.4)
Organ support on the first day of critical care unit								
Vasopressor use	1515 (18.2)	2684 (22.8)	6209 (27.0)	11,448 (29.8)	26,733 (30.8)	39,788 (28.0)	37,178 (23.6)	7150 (16.4)
MV	2157 (25.9)	3053 (25.9)	5910 (25.7)	9736 (25.3)	22,288 (25.6)	34,208 (24.1)	30,572 (19.4)	5457 (12.5)
RRT	423 (5.1)	800 (6.8)	1976 (8.6)	3567 (9.3)	8039 (9.2)	10,383 (7.3)	6928 (4.4)	691 (1.6)
Comorbidities								
CHF	645 (7.8)	1141 (9.7)	2589 (11.2)	4556 (11.9)	12,250 (14.1)	25,068 (17.6)	36,248 (23.0)	12,656 (29.0)
Diabetes	412 (5.0)	1207 (10.2)	4087 (17.7)	8622 (22.4)	21,574 (24.8)	33,641 (23.7)	28,301 (18.0)	4966 (11.4)
Renal failure	142 (1.7)	464 (3.9)	1518 (6.6)	3481 (9.1)	9013 (10.4)	13,752 (9.7)	12,500 (7.9)	2645 (6.1)
Liver disease	357 (4.3)	777 (6.6)	1937 (8.4)	3654 (9.5)	6349 (7.3)	7241 (5.1)	5532 (3.5)	756 (1.7)
AIDS/HIV	53 (0.6)	122 (1.0)	189 (0.8)	109 (0.3)	112 (0.1)	58 (0.0)	23 (0.0)	0 (0.0)
Lymphoma	93 (1.1)	152 (1.3)	338 (1.5)	693 (1.8)	1930 (2.2)	2547 (1.8)	1622 (1.0)	190 (0.4)
Cancer/tumor	260 (3.1)	571 (4.8)	1877 (8.1)	4736 (12.3)	14,276 (16.4)	23,085 (16.2)	18,729 (11.9)	3349 (7.7)
Obesity	53 (0.6)	120 (1.0)	189 (0.8)	142 (0.4)	135 (0.2)	102 (0.1)	57 (0.0)	7 (0.0)
Weight loss	20 (0.2)	38 (0.3)	103 (0.4)	189 (0.5)	361 (0.4)	459 (0.3)	482 (0.3)	146 (0.3)
Focus of infections								
Respiratory	3544 (42.6)	4592 (38.9)	8506 (36.9)	14,434 (37.6)	35,768 (41.1)	64,147 (45.1)	74,977 (47.6)	22,384 (51.2)
Abdominal	1735 (20.8)	2671 (22.7)	5829 (25.3)	10,482 (27.3)	23,852 (27.4)	37,104 (26.1)	39,958 (25.4)	10,153 (23.2)
Urogenital	499 (6.0)	729 (6.2)	1461 (6.3)	2515 (6.5)	6217 (7.2)	13,396 (9.4)	20,496 (13.0)	7717 (17.7)
Cardiovascular	916 (11.0)	1293 (11.0)	2331 (10.1)	3193 (8.3)	6129 (7.1)	8495 (6.0)	7388 (4.7)	1450 (3.3)
CNS	1697 (20.4)	1836 (15.6)	2594 (11.3)	3387 (8.8)	5681 (6.5)	7071 (5.0)	5511 (3.5)	861 (2.0)
Blood	510 (6.1)	829 (7.0)	1618 (7.0)	2537 (6.6)	5025 (5.8)	7121 (5.0)	6354 (4.0)	1294 (3.0)
Bone & soft tissue	463 (5.6)	878 (7.4)	1892 (8.2)	2784 (7.2)	4682 (5.4)	5757 (4.1)	5594 (3.5)	1537 (3.5)
Undetected/NA	945 (11.4)	1561 (13.2)	3536 (15.4)	6510 (16.9)	14,766 (17.0)	22,878 (16.1)	23,419 (14.9)	5607 (12.8)

Data is presented as numbers with percentages or medians with interquartile ranges (IQRs). *BMI* = body mass index, *ICU* = intensive care unit, *HDU* = high dependency unit, *EICU* = emergency intensive care unit, *MV* = mechanical ventilation, *RRT* = renal replacement therapy, *CHF* = congestive heart failure, *AIDS/HIV* = acquired immunodeficiency syndrome/human immunodeficiency virus, *CNS* = central nerve system, *NA* = not available

^a^Elixhauser score stands for Elixhauser comorbidity score

^b^Surgical patients refer to those performed under general anesthesia during hospitalization

### Clinical outcomes

Mortality rates increased progressively with age. Critical care unit mortality rose from 3.6% in patients aged ≤ 29 years to 9.5% in those aged ≥ 90 years, while hospital mortality increased from 8.3% to 24.9% across the same age groups (Table 2). In unadjusted analysis, patients aged ≥ 90 years had an odds ratio (OR) of 3.68 (95% CI 3.39–3.99) for in-hospital mortality compared to the 18–29-year reference group. Generalized linear mixed effects models confirmed that advanced age remained significantly associated with higher mortality after adjustment. In Model 1 (adjusted for sex, comorbidity, and hospital-level clustering) the OR for patients ≥ 90 years was 3.96 (95% CI 3.65–4.30). In Model 2, which further adjusted for vasopressor use, mechanical ventilation, and RRT on the day of admission, the OR increased to 4.75 (95% CI 4.37–5.16). ORs for younger age groups were attenuated in the same model (Table 3).

**Table 2:** Clinical outcomes

	18–29 N = 8,322	30–39 N = 11,790	40–49 N = 23,033	50–59 N = 38,410	60–69 N = 86, 928	70–79 N = 142,083	80–89 N = 15,7588	90 + N = 43,694
Hospital mortality	687 (8.3)	1171 (9.9)	2887 (12.5)	6124 (15.9)	16,596 (19.1)	31,270 (22.0)	37,837 (24.0)	10,859 (24.9)
Critical care unit mortality	298 (3.6)	536 (4.5)	1293 (5.6)	2507 (6.5)	6629 (7.6)	11,808 (8.3)	14,467 (9.2)	4139 (9.5)
Hospital LOS	16 [9, 37]	18 [9, 38]	20 [11, 42]	21 [12, 43]	22 [12, 43]	22 [12, 41]	21 [11, 38]	18 [10, 32]
Critical care unit LOS	4 [2, 9]	4 [2, 9]	4 [2, 9]	4 [2, 9]	4 [2, 9]	4 [2, 8]	4 [2, 7]	3 [2, 6]
Organ support rates during critical care unit stay								
Vasopressor use	2236 (26.9)	3757 (31.9)	8452 (36.7)	15,367 (40.0)	35,578 (40.9)	53,065 (37.3)	49,156 (31.2)	9324 (21.3)
MV	3184 (38.3)	4521 (38.3)	9052 (39.3)	15,229 (39.6)	34,585 (39.8)	52,228 (36.8)	46,425 (29.5)	8178 (18.7)
RRT	796 (9.6)	1592 (13.5)	3733 (16.2)	6823 (17.8)	15,663 (18.0)	20,659 (14.5)	14,485 (9.2)	1482 (3.4)
Organ support days after critical care unit admission^a^								
Vasopressor use	3 [2, 6]	3 [2, 6]	3 [2, 6]	3 [2, 6]	3 [2, 6]	3 [2, 6]	3 [2, 5]	3 [1, 4]
MV	8 [3, 17]	7 [3, 15]	6 [3, 13]	6 [3, 13]	6 [3, 14]	6 [3, 14]	5 [2, 12]	4 [2, 9]
RRT	7 [3, 14]	7 [3, 14]	6 [3, 14]	7 [3, 14]	6 [3, 14]	6 [3, 14]	5 [2, 11]	4 [2, 8]

Data is presented as numbers with percentages or medians with interquartile ranges (IQRs). *LOS* = length of stay, *MV* = mechanical ventilation, *RRT* = renal replacement therapy

^a^Organ support days after critical care admission were calculated for the population requiring each of the three organ support modalities

**Table 3:** Association between age group and hospital mortality

Age group	Unadjusted model Odds ratio (95% CI)	Adjusted model 1^a^ Odds ratio (95% CI)	Adjusted model 2^b^ Odds ratio (95% CI)
18–29	Reference	Reference	Reference
30–39	1.23 (1.11–1.35)	1.20 (1.08–1.32)	1.15 (1.04–1.27)
40–49	1.59 (1.46–1.74)	1.51 (1.38–1.65)	1.40 (1.28–1.54)
50–59	2.11 (1.94–2.29)	1.94 (1.79–2.11)	1.80 (1.65–1.96)
60–69	2.62 (2.42–2.84)	2.41 (2.22–2.61)	2.23 (2.05–2.42)
70–79	3.14 (2.90–3.39)	2.95 (2.73–3.20)	2.88 (2.65–3.12)
80–89	3.51 (3.25–3.80)	3.50 (3.23–3.79)	3.72 (3.43–4.04)
90 +	3.68 (3.39–3.99)	3.96 (3.65–4.30)	4.75 (4.37–5.16)

^a^Adjusted logistic regression model 1: Covariates included sex and the Elixhauser comorbidity score as fixed effects, while hospital was treated as a random effect to account for baseline differences

^b^Adjusted model 2: Additionally incorporated vasopressor use, mechanical ventilation, and RRT on the day of critical care unit admission as surrogate markers for disease severity

**Fig. 3:**
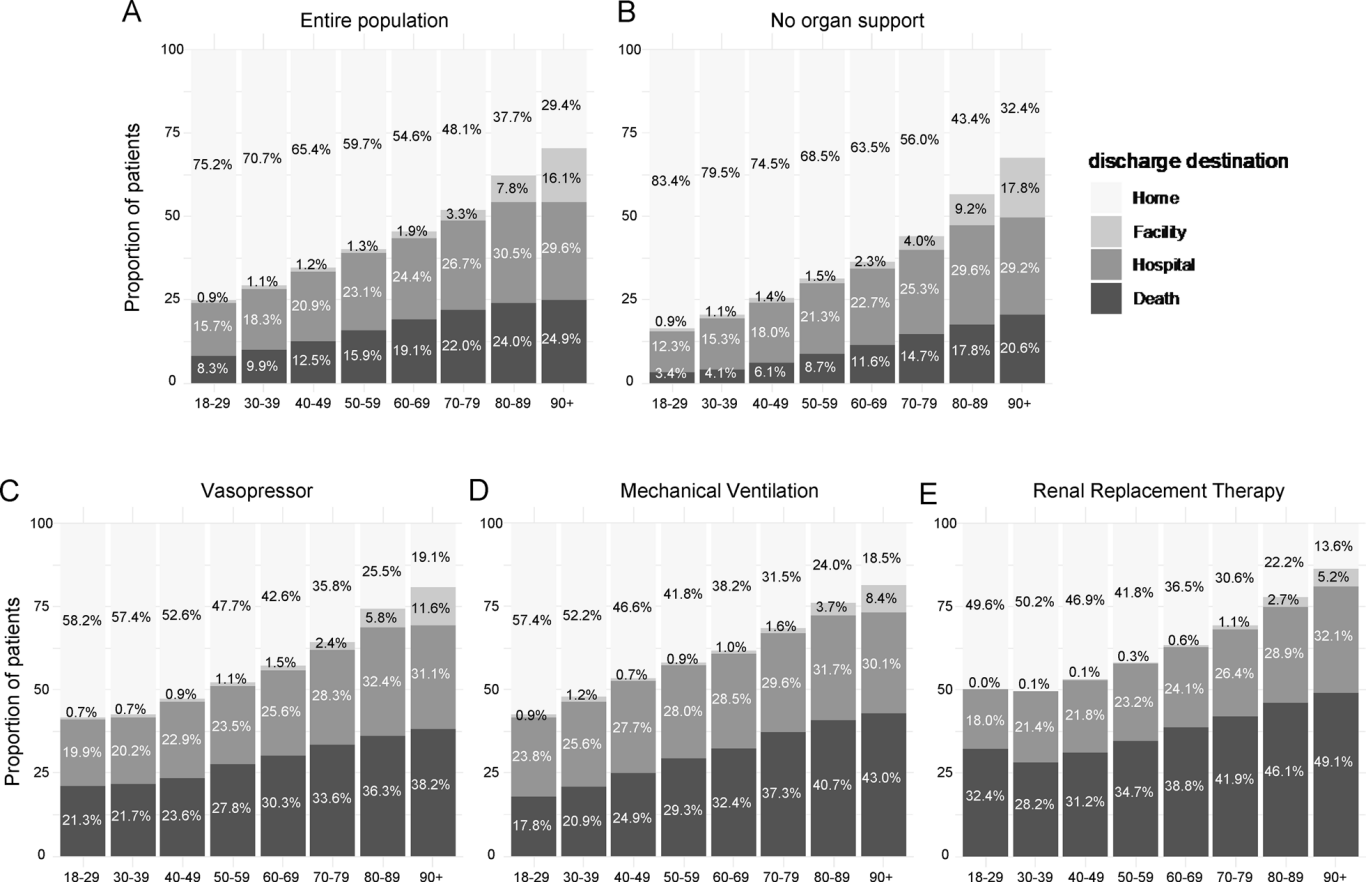
Discharge destinations across age groups. Each panel shows discharge destinations according to the type of organ support received on the first day in the critical care unit: **A** entire cohort regardless of organ support; **B** patients without any organ support; **C** patients supported with vasopressors; **D** patients supported with mechanical ventilation; and **E** patients supported with renal replacement therapy

Hospital length of stay remained relatively stable until around 80 years of age, after which it declined with increasing age. Organ support utilization peaked in the 60–69 age group and decreased among older age groups (Table 2). The proportion of patients discharged home declined sharply with age, falling from 75.2% in patients aged ≤ 29 years to 29.4% in those aged ≥ 90 years. Transfer to another hospital was most frequent among patients aged 80–89 years (30.5%), while discharge to nursing-care facilities was highest among patients aged ≥ 90 years (16.1%). Among patients who received organ support therapy, both mortality rates and likelihood of home discharge worsened with increasing age, except for a small subgroup of very young patients undergoing RRT. Compared with those who did not receive organ support, all subpopulations requiring organ support had higher mortality and lower home discharge rates. Notably, patients requiring RRT had the highest mortality and lowest home discharge rates, suggesting particularly poor outcomes in this subgroup. Further data are presented in Fig. 3 and Supplementary Table S3.

## Discussion

Using a nationwide database, this study provides a comprehensive description of sepsis epidemiology among patients admitted to critical care units in Japan. Patients aged 80–89 years constituted the largest subgroups of sepsis cases, and those aged ≥ 70 years accounted for approximately two-thirds of the cohort, a proportion that increased steadily over the study period. Respiratory infections were the most prevalent source of sepsis across all age groups, followed by intra-abdominal infections. We observed clear age-related differences in infection sources: urinary tract infections were more common among older patients, whereas CNS infections were more frequent among younger patients. Notably, older age groups had substantially higher ICU and hospital mortality and were less likely to be discharged home. Interestingly, the 60–69-year age group demonstrated the highest use of organ support therapies, and the longest hospital stays compared with other age groups.

The high proportion of elderly patients with sepsis in critical care units likely reflects Japan’s unique demographic and healthcare context. First, patients aged ≥ 70 years accounted for 67% of the study cohort, increasing over the 10-year study period, a proportion much higher than that reported in other countries [2, 17, 23]. This difference may be attributed to Japan’s demographic changing —with 14% of its population is over 75 years —as well as characteristics of its healthcare system that facilitate access to intensive care use among elderly individuals [24, 25]. While the observed increase in the proportion of elderly septic patients is likely a consequence of the national demographic trend of population aging, this finding remains clinically important as it quantifies the growing burden of elderly patients with sepsis in Japanese critical care settings. Japan’s universal health insurance system minimizes out-of-pocket expenses, particularly for the elderly, potentially encouraging greater use of acute care services [26]. Additionally, the limited uptake of advance care planning in Japan may contribute to high utilization of intensive care among elderly patients at end of life [27]. These socio-cultural and systemic factors are insufficiently studied and warrant further investigation.

Second, the study highlights distinct trends in resource utilization and treatment approaches among patients ≥ 90 years. This group was less likely to receive advanced interventions, particularly mechanical ventilation, compared to younger patients — a trend consistent with a recent Japanese ICU registry study [28]. The lower use of advanced therapies in this population may reflect clinical decisions to forgo intensive treatments based on perceived futility or potential harm. This interpretation is supported by our findings that a larger proportion of patients ≥ 90 years were treated in EICUs or HDUs, which provide lower-intensity care than conventional ICUs.

Advancing age independently increased the risk of in-hospital mortality, highlighting age-related vulnerability as a key determinant of patients’ outcomes following sepsis. This is consistent with previous studies identifying age as a major risk factor for sepsis-related death [1, 17]. Frailty may partially explain the higher mortality and lower home discharge rates observed in older patients, although our dataset did not include direct frailty assessment [10, 11, 14]. In addition, social factors such as isolation or delays in seeking care may also negatively impact sepsis outcomes in elderly populations and warrant further investigation.

Our findings underscore substantial outcome variation based on organ support use, especially in older adults. Patients who received organ support on the first ICU day had higher mortality and were less often discharged home. This aligns with a prior Japanese study showing poor long-term outcomes among elderly patients receiving mechanical ventilation [14]. Previously, eICU-Database which represented multi-center ICU from 208 hospitals across the USA illustrated ICU-mortality was higher in the very old patients (≥ 80 years) than old patients (65–79 years), while very old patients supported by mechanically ventilation were significantly less often than the old group [29]. Notably, in our study, the adjusted odds ratio for hospital mortality among patients ≥ 90 years rose considerably when organ support was accounted for, whereas estimates for younger groups changed little. This suggests that very elderly patients face high mortality regardless of support intensity, possibly due to limited physiological reserve. Further research is needed to identify which older patients might still benefit from invasive interventions. This study represents one of the largest and most comprehensive investigations to date on the age-related epidemiology of sepsis in a super-aged society. By focusing specifically on patients with sepsis, we minimized population heterogeneity compared to broader ICU-based studies [28]. Moreover, we incorporated data from a wide spectrum of critical care settings, including ICUs, HDUs and EICUs, offering a broad and realistic perspective on sepsis management in Japan. A further strength is that our study cohort reflects future global demographic trends: by 2050, more than 20% of the global population is projected to be aged ≥ 60 years [3, 30–34]. Japan reached this threshold in 2000, with individuals ≥ 60 years comprising 30–35% of the population during the study period [25]. To our knowledge, this is the most extensive description to date of sepsis care among patients aged ≥ 90 years, providing valuable insights for healthcare policy and resource planning in aging society. Our findings underscore the importance of developing age-appropriate critical care strategies and policies as the global population continues to age.

Several limitations warrant mention. First, the DPC database is designed primarily for administrative and billing purposes rather than clinical research. As a result, it lacks detailed clinical information such as severity scores and laboratory data, limiting the precision of outcome assessment. We attempted to mitigate this limitation by using organ support variables as surrogate markers of disease severity, although residual confounding is likely. Moreover, while the DPC database does not specify the triggering cause of ICU/HDU/EICU admission, our cohort definition required not only an infection-related diagnosis but also concurrent IV antibiotic administration and blood culture testing within a 3-day window of admission. This combination strongly suggests that a suspected septic condition was present and likely represented the primary reason for the critical care unit admission. Second, we defined sepsis using infection-related ICD-10 codes, antibiotic administration, and blood culture testing, rather than the Sepsis-3 criteria, which could not be applied due to insufficient data within our DPC dataset [35]. This approach may have introduced selection bias by including patients with milder infections or excluding those who met Sepsis-3 definitions without appropriate coding. Furthermore, we did not include “organ dysfunction” or “organ support” codes in the inclusion criteria for the following reasons: the DPC database may undercapture organ dysfunction because only a limited number of diagnostic codes are recorded at discharge; the use of organ support codes could be confounded by end-of-life care decisions, particularly among older patients; and temporal information to link organ dysfunction to sepsis is unavailable in the dataset [36]. Nonetheless, our sepsis definition, requiring three specific elements, ensured a highly specific cohort of patients with severe infection. Furthermore, by focusing on patients admitted to a critical care unit (ICU/HDU/EICU), we tried targeting a population with a more severe illness and organ dysfunction, providing a robust proxy for patients meeting the Sepsis-3 criteria. Third, we were unable to capture post-discharge outcomes, as the DPC database includes data only during acute care hospitalization. This limitation may have resulted in the censoring of patients who died after transfer to non-acute hospitals, nursing facilities, or hospice care. However, more than 75% of patients in our cohort were hospitalized for over 30 days. Suggesting that our findings likely reflect outcomes during the acute phase of illness in most elderly patients with sepsis. Fourth, the generalizability of our findings may be limited due to Japan-specific healthcare structure, ICU practice, and cultural attitudes toward end-of-life care, which may differ from those in other countries. Especially, during time period, several guidelines and treatment strategies were altered such as initial resuscitation for sepsis and RRT indication for acute kidney injury due to sepsis. Furthermore, we could not capture the presence or absence of treatment-limiting decisions regarding end-of-life care, which might be related to resource utilization and mortality. These influences should be warrants consideration to interpret the results. Finally, our dataset ends in March 2020 and does not capture changes related to the COVID-19 pandemic. While this may be viewed as a limitation, it also serves as a strength by a stable, pre-pandemic baseline free from the confounding effects of pandemic-related healthcare system disruptions.

## Conclusions

We identified marked age-related variations in the epidemiology, clinical outcomes, and resource utilization of sepsis in Japanese critical care units. The very elderly population (≥ 80 years) accounted for nearly 40% of the cohort, and mortality rates rose sharply with advancing age. Only 37.7% of patients in their 80 s and 29.4% of those aged ≥ 90 years were discharged home. These findings underscore the clinical and logistical challenges of managing sepsis in an aging society and highlight the need for strategic planning and resource allocation. As global populations continue to age, the insights from this study provide valuable evidence to inform sepsis management and healthcare policies in elderly population worldwide.

## Supplementary Information

Supplementary material 1.

## Data Availability

The datasets analyzed in this study are not publicly available to protect patient confidentiality.
