# Supplementary material for: Age-related epidemiology and outcomes of sepsis in Japanese critical care units: a nationwide administrative claims database study
Source: J Intensive Care. 2025 Dec 17;13:66. doi: 10.1186/s40560-025-00837-4 (PMC12709830; doi:10.1186/s40560-025-00837-4)
Supplement: Supplementary file 1 — Supplementary material 1. [file 40560_2025_837_MOESM1_ESM.pdf]

Supplementary Materials: Table of Contents

Supplementary File 1. Code lists

List 1. International classification of disease version 10 (ICD-10) codes for infectious disease ………..…………………….. P2

List 2. Critical care unit codes ……..…………………………………………………………………………….. P6

List 3. Procedure codes ………………………………………………………………………………………… P8

List 4. Antimicrobial codes ………………………………………………………………………………………… P9

List 5. Vasopressor codes ………………………………………………………………………………………… P20

Supplementary File 2. Supplementary tables

Table S1. Background of patients, overall cohort ………………………………………...………………….. P21

Table S2. Comorbidities ………………………………………………………………………….…………………... P22

Table S3 Discharge destination by organ support type on the day of critical care unit admission ………………………………… P24

Supplementary file 1. Code lists

List 1. International classification of disease version 10 (ICD-10) codes for infectious disease

ICD-10 codes
A00, A000, A001, A009, A01, A010, A011, A012, A013, A014, A02, A020, A021, A022, A028, A029, A03, A030, A031, A032, A033, A038, A039, A04, A040, A041, A042, A043, A044, A045, A046, A047, A048, A049, A05, A050, A051, A052, A053, A054, A058, A059, A06, A060, A061, A062, A063, A064, A065, A066, A067, A068, A069, A07, A070, A071, A072, A073, A078, A079, A08, A080, A081, A082, A083, A084, A085, A085a, A085b, A09, A15, A150, A151, A152, A153, A154, A155, A156, A157, A158, A159, A16, A160, A161, A162, A163, A164, A165, A167, A168, A169, A17, A170, A171, A178, A179, A18, A180, A181, A182, A183, A184, A185, A186, A187, A188, A19, A190, A191, A192, A198, A199, A20, A200, A201, A202, A203, A207, A208, A209, A21, A210, A211, A212, A213, A217, A218, A219, A22, A220, A221, A222, A227, A228, A229, A23, A230, A231, A232, A233, A238, A239, A24, A240, A241, A242, A243, A244, A25, A250, A251, A259, A26, A260, A267, A268, A269, A27, A270, A278, A279, A28, A280, A281, A282, A288, A289, A30, A300, A301, A302, A303, A304, A305, A308, A309, A31, A310, A311, A318, A319, A32, A320, A321, A327, A328, A329, A33, A34, A35, A36, A360, A361, A362, A363, A368, A369, A37, A370, A371, A378, A379, A38, A39, A390, A391, A392, A393, A394, A395, A398, A399, A40, A400, A401, A402, A403, A408, A409, A41, A410, A411, A412, A413, A414, A415, A418, A419, A42, A420, A421, A422, A427, A428, A429, A43, A430, A431, A438, A439, A44, A440, A441, A448, A449, A46, A48, A480, A481, A482, A483, A484, A488, A49, A490, A491, A492, A493, A498, A499, A50, A500, A501, A502, A509, A51, A510, A511, A512, A514, A515, A519, A52, A520, A521, A523, A527, A53, A530, A539,

A54, A540, A541, A542, A544, A545, A546, A548, A549, A55, A56, A560, A561, A562, A563, A564, A568, A59, A590, A598, A599, A60, A600, A601, A609, A63, A638, A64, A65, A66, A660, A667, A669, A68, A680, A681, A689, A69, A690, A691, A692, A698, A699, A70, A74, A748, A749, A75, A750, A751, A752, A753, A759, A77, A770, A771, A772, A773, A778, A778a, A778b, A779, A78, A79, A790, A791, A798, A799, A80, A800, A801, A802, A803, A804, A809, A81, A810, A811, A812, A818, A819, A82, A820, A821, A829, A83, A830, A831, A832, A833, A834, A835, A836, A838, A839, A84, A840, A841, A848, A849, A85, A850, A851, A852, A858, A86, A87, A870, A871, A872, A878, A879, A88, A880, A888, A89, A90, A91, A92, A920, A921, A922, A923, A924, A928, A929, A93, A930, A931, A932, A938, A94, A95, A950, A951, A959, A96, A960, A961, A962, A968, A969, A98, A980, A981, A982, A983, A984, A985, A988, A99, B00, B001, B002, B003, B004, B005, B007, B008, B009, B01, B010, B011, B012, B018, B019, B02, B020, B021, B022, B023, B027, B028, B029, B03, B04, B05, B050, B051, B052, B053, B054, B058, B059, B06, B060, B068, B069, B08, B080, B082, B083, B084, B085, B088, B09, B15, B150, B159, B16, B160, B161, B162, B169, B17, B170, B171, B172, B178, B18, B180, B181, B182, B188, B189, B19, B190, B199, B20, B200, B201, B202, B203, B204, B205, B206, B207, B208, B209, B22, B23, B230, B238, B24, B25, B250, B251, B252, B258, B259, B26, B260, B261, B262, B263, B268, B269, B27, B270, B271, B278, B279, B30, B300, B301, B302, B303, B308, B309, B33, B330, B331, B332, B333, B334, B338, B34, B340, B341, B342, B343, B344, B348, B349, B37, B371, B374, B375, B376, B377, B378, B379, B38, B380, B381, B382, B384, B387, B388, B389, B39, B390, B391, B392, B393, B394, B395, B399, B40, B400, B401, B402, B407, B408, B409, B41, B410, B417, B418, B419, B42, B420, B421, B427, B428, B429, B43, B431, B432, B438, B439, B44, B440, B441, B442, B447, B448, B449, B45, B450, B451, B453, B457, B458, B459, B46, B460, B461, B462, B464, B465, B468, B469, B48, B482, B483,

B484, B487, B488, B49, B50, B500, B508, B509, B51, B510, B518, B519, B52, B520, B528, B529, B53, B530, B531, B538, B54, B55, B550, B559, B56, B560, B561, B569, B57, B570, B571, B572, B573, B574, B575, B58, B581, B582, B583, B588, B589, B59, B60, B600, B601, B602, B608, B64, B65, B650, B651, B652, B658, B659, B66, B660, B661, B662, B663, B664, B665, B668, B669, B67, B670, B671, B672, B673, B674, B675, B676, B677, B678, B679, B68, B680, B681, B689, B69, B690, B698, B699, B70, B700, B701, B71, B710, B711, B718, B719, B72, B73, B74, B740, B741, B742, B743, B744, B748, B749, B75, B76, B760, B761, B768, B769, B77, B770, B778, B779, B78, B780, B781, B787, B789, B79, B81, B811, B813, B814, B818, B82, B820, B829, B83, B830, B831, B832, B833, B838, B839, B89, B95, B950, B951, B952, B953, B954, B955, B956, B957, B958, B96, B960, B961, B962, B963, B964, B965, B966, B967, B968, B97, B970, B971, B972, B973, B974, B975, B976, B977, B978, B99, D733, E321, G00, G000, G001, G002, G003, G008, G009, G01, G02, G020, G021, G028, G03, G031, G032, G038, G039, G04, G040, G042, G048, G049, G05, G050, G051, G052, G058, G06, G060, G061, G062, G07, G08, H050, H60, H600, H601, H602, H603, H608, H609, H610, H620, H621, H622, H623, H650, H652, H653, H66, H660, H661, H662, H663, H664, H669, H670, H671, H68, H680, H700, H701, H702, H708, H709, H730, H731, H750, H830, I30, I301, I308, I309, I320, I321, I33, I330, I339, I38, I40, I400, I401, I408, I409, I410, I411, I412, I430, I520, I521, I681, I80, I800, I801, I802, I803, I808, I809, I821, I891, I980, I981, J00, J01, J010, J011, J012, J013, J014, J018, J019, J02, J020, J028, J029, J03, J030, J038, J039, J04, J040, J041, J042, J05, J050, J051, J06, J060, J068, J069, J10, J100, J101, J108, J11, J110, J111, J118, J12, J120, J121, J122, J128, J129, J13, J14, J15, J150, J151, J152, J153, J154, J155, J156, J157, J158, J159, J16, J160, J168, J17, J170, J171, J172, J173, J178, J18, J180, J181, J182, J188, J189, J20, J200, J201, J202, J203, J204, J205, J206, J207, J208, J209, J21, J210, J218, J219, J22, J31, J310, J311, J312, J32, J320, J321, J322, J323, J324,

J328, J329, J340, J350, J36, J37, J370, J371, J390, J391, J40, J41, J410, J411, J418, J42, J85, J851, J852, J853, J86, J860, J869,

K040, K046, K047, K103, K112, K113, K122, K35, K350, K351, K359, K36, K37, K515, K570, K572, K574, K578, K603, K604,

K605, K61, K610, K611, K612, K613, K614, K630, K631, K65, K650, K658, K659, K67, K670, K671, K672, K673, K678, K750,

K751, K770, K800, K801, K803, K804, K81, K810, K811, K818, K819, K822, K823, K830, K832, K833, L00, L01, L010, L011,

L02, L020, L021, L022, L023, L024, L028, L029, L03, L030, L031, L032, L033, L038, L039, L04, L040, L041, L042, L043, L048,

L049, L050, L08, L080, L088, L089, L303, L663, L732, L88, L980, M00, M000, M001, M002, M008, M009, M01, M010, M011,

M012, M013, M014, M015, M016, M018, M462, M463, M465, M490, M491, M492, M493, M600, M630, M631, M632, M650,

M651, M680, M710, M711, M726, M730, M731, M86, M860, M861, M862, M863, M864, M865, M866, M868, M869, M900,

M901, M902, N110, N111, N136, N151, N30, N300, N330, N340, N390, N410, N411, N412, N413, N431, N45, N450, N459, N70,

N700, N701, N709, N71, N710, N711, N719, N72, N73, N730, N731, N732, N733, N734, N735, N736, N738, N739, N74, N740,

N741, N742, N743, N744, N748, N751, N760, N761, N762, N763, N764, N770, N771, O030, O035, O040, O045, O050, O055,

O060, O065, O070, O075, O080, O222, O23, O230, O231, O232, O233, O234, O235, O239, O740, O753, O85, O86, O860, O861,

O862, O863, O868, O870, O883, O91, O910, O911, O98, O980, O981, O982, O983, O984, O985, O986, O988, O989, P23, P230,

P231, P232, P233, P234, P235, P236, P238, P239, P35, P350, P351, P352, P353, P358, P359, P36, P360, P361, P362, P363, P364,

P365, P368, P369, P37, P370, P371, P372, P373, P374, P375, P378, P379, P39, P390, P392, P393, P394, P398, P399, P780, P781,

R091, T793, T802, T814, T826, T827, T835, T836, T845, T846, T847, T857, T874, T880, U04, U049

List 2. Critical care unit codes

Types of critical care unit	specific medical codes for reimbursement
intensive care unit	190174510, 190174410, 190174710, 190174610, 190174910, 190174810, 190116310, 190116410, 190139810, 190140010, 190140110, 190139910, 193005910, 193006410, 193010410, 193010710, 193010310, 193001610, 193010010, 193010110, 193010210, 193006210, 193001710, 193010610, 193010510, 193006110, 193006310, 193006010, 193507810, 193511810, 193511710, 193501610, 193501710, 193512010, 193508010, 193507910, 193512410, 193507610, 193507710, 193512310, 193512210, 193512110, 193507510, 193511910, 193307510, 193301510, 193310810, 193307410, 193307710, 193310410, 193310510, 193310710, 193310310, 193310610, 193307610, 193310210, 193310910, 193301410, 193307310, 193307210
high dependency unit	190175010, 190175110, 193011110, 193010810, 193011010, 193010910, 193512510, 193512810, 193512610, 193512710, 193311010, 193311310, 193311210, 193311110, 190117310, 193003110, 193003010, 193501910, 193501810, 193301810, 193301910

emergency intensive care unit	190128610, 190024510, 190074510, 190128710, 190024310, 190024410, 190138510, 190138610, 190138310, 190138210, 190138410, 190138110, 190138710, 190138810, 190139310, 190139210, 190138910, 190139110, 190139010, 193004810, 193005110, 193000910, 193000610, 193004610, 193005010, 193003910, 193005410, 193003310, 193004010, 193004210, 193001210, 193004410, 193000310, 193004910, 193004110, 193004510, 193003410, 193005210, 193004710, 193004310, 193005310, 193505810, 193506610, 193500910, 193506010, 193511570, 193511670, 193506810, 193506410, 193506210, 193505510, 193504710, 193501210, 193506910, 193507010, 193505710, 193506510, 193506710, 193506110, 193505910, 193504610, 193500310, 193500610, 193505610, 193506310, 193306710, 193306610, 193306410, 193300110, 193306010, 193305710, 193305810, 193305410, 193304410, 193306210, 193306110, 193305210, 193300710, 193300410, 193305910, 193304310, 193306510, 193306310, 193305610, 193305510, 193305310, 193301010

List 3. Procedure codes

Types of procedures	specific medical codes for reimbursement
Blood culture test	160058610
Ventilator support	140009310, 140023510, 140010150, 140024350
Renal replacement therapy	140051110, 140051010, 140036710, 140052810, 140007710, 140029850

List 4. Antimicrobial codes

Types of antimicrobials	specific medical codes for reimbursement
antibacterial agent	620005060, 640463089, 620000427, 620006026, 620006027, 620006165, 620007537, 622329900, 621062901, 620006341, 620007538, 621380904, 621380908, 622330000, 620006342, 622329800, 621977300, 620006344, 620006343, 620006224, 620006261, 620006348, 620006348, 620008810, 621064601, 622111302, 621064701, 621064501, 620006225, 620006262, 620006349, 620006349, 620008811, 621065101, 621065201, 621065301, 621065001, 620006226, 620006227, 620003894, 620003894, 620003894, 620006791, 620008047, 620009408, 621738802, 640463091, 640463091, 620002907, 620002907, 620005694, 620005695, 620005695, 620006792, 620009575, 620009576, 621487601, 621487701, 621487802, 622107901, 622107901, 621942602, 621971901, 620004729, 620009511, 621876001, 621735001, 620004730, 620004730, 620009512, 621066501, 621066401, 620004728, 620009509, 621924501, 621896701, 620007432, 621955601, 621950502, 621936302, 620004709, 620004709, 620008212, 620008213, 620008532, 620008532, 620008531, 620008531, 620008533, 620008534, 620008534, 620008535, 620009572, 620009572, 620009571, 621995201, 622227801, 621901402, 621884501, 620009574, 620009574, 622216601, 622207201, 622227901, 622338101, 622085501, 640462005, 640462005, 620006477, 646130274, 646130275, 620008598, 620008599, 620008600, 640453139, 640453140, 646120062, 620003676, 621069302, 621069501,

	622326000, 620003190, 621069402, 621069001, 620003462, 620003677, 621070102, 621070301, 620003191, 621070202, 621069701, 621070602, 621070902, 640453078, 620003198, 620003193, 620005641, 621604701, 646120080, 646120081, 640453079, 620005642, 621695601, 622213301, 620009196, 620008618, 620008753, 620008752, 622415701, 620003657, 620003658, 616120011, 616120012, 622199101, 610454003, 620006920, 620006920, 620007026, 620007026, 620007026, 622066601, 622112902, 620005906, 621073001, 616130132, 616130295, 616130039, 620007024, 622054901, 622066501, 622111101, 622127801, 622165902, 616130040, 620006829, 620006829, 620006829, 620006919, 620007025, 620008584, 620009117, 621073901, 621074701, 621075101, 621075101, 610451023, 621076301, 621076401, 621076401, 621076601, 621076801, 621077201, 621077501, 646130136, 620007456, 621078403, 621078301, 621078103, 621078601, 621342601, 646130137, 620007457, 621079203, 621079101, 621078903, 621079401, 621342802, 640462059, 622079701, 640462060, 622079801, 616130002, 621083104, 622316900, 616130378, 616130378, 616130003, 620003645, 621083503, 610454041, 610454042, 620004512, 620004981, 610453048, 610454043, 620003646, 616130115, 616130229, 620003647, 621085602, 621085602, 621087903, 622316800, 616130112, 621086806, 621087001, 621088403, 616130469, 616130110, 620008657, 621088802, 621089701, 621089501, 621088705, 621089103, 621089103, 621088901, 620005947, 616130404, 616130404, 616130404, 616130404, 610453059, 620000061, 616130405, 616130405,

	616130405, 616130406, 616130406, 616130406, 620002756, 620002756, 620003583, 621675801, 616130407, 616130407, 616130408, 616130408, 620004958, 620004958, 620003968, 620003968, 620003968, 620003968, 620003970, 620008681, 620008681, 620008681, 621744203, 621744401, 621744401, 621747601, 620008682, 620008682, 621747701, 616130476, 616130476, 616130477, 620004513, 620004513, 621935801, 620008481, 620008483, 620008483, 620008483, 620008483, 620008482, 620008482, 620008482, 620008484, 620009343, 620009343, 620009344, 621889403, 621885302, 621935901, 620008487, 620008488, 620008489, 620008490, 616130531, 620008491, 620009334, 620009335, 620009335, 620009337, 620009337, 620009336, 621910002, 621885102, 616130532, 616130532, 620008492, 620009339, 620009340, 620009340, 620009342, 620009342, 620009342, 620009341, 621910102, 621885202, 620005503, 620005503, 620005501, 620005502, 620005499, 620005499, 620005500, 620008746, 620004080, 620005506, 620005506, 620005505, 620005507, 620005508, 610411059, 621955901, 621939401, 621940201, 621940201, 621940201, 621940201, 621940201, 621946301, 621946301, 621946301, 621931001, 621931001, 621962401, 621962501, 621942904, 610411057, 620009331, 621956001, 621956001, 621930801, 621940301, 621947501, 621947501, 621962601, 621943004, 610411058, 620009332, 621956101, 621956101, 621930901, 621940401, 621940401, 621940401, 621940401, 621947601, 621947601, 621947601, 621962701, 621943104, 646130121, 646130110, 646130122, 620004140, 620007293, 620007293,

	620007293, 621989702, 621987602, 621924001, 646130111, 646130123, 620004141, 620007294,<br>620007294, 620007294, 620007294, 621989802, 621344901, 621924101, 646130112, 646130124,<br>620003732, 620007295, 620007295, 620007295, 620007295, 621345002, 621345301, 621095002,<br>621095002, 640408149, 620009563, 621540002, 640408148, 620003735, 622104201, 622130801,<br>622177601, 622124902, 622033402, 622104301, 622177701, 622328100, 620003736, 621096002,<br>620003737, 621096701, 621096503, 621096802, 621096601, 620003738, 621097303, 622177801,<br>621097602, 621097401, 620003733, 620003734, 620003740, 620003739, 620007318, 621757001,<br>621757001, 620005674, 620005675, 646130072, 646130073, 622125001, 622104001, 622131001,<br>622111501, 646130074, 621987501, 622104101, 622131101, 622111601, 646130075, 620006243,<br>620007540, 621101802, 621102102, 646130076, 620006244, 620007541, 621101902, 621102802,<br>640470010, 620004151, 621951701, 640454018, 620004652, 640454019, 620004653, 646130067,<br>646130067, 646130071, 646130132, 646130133, 646130134, 620002999, 620002999, 620002999,<br>620002999, 620002999, 620003827, 620006701, 620006701, 621967001, 621936401, 622052901,<br>622124801, 621756202, 621755802, 621755802, 621755802, 620003000, 620003000, 620003000,<br>620003000, 620003000, 620003001, 620003828, 620005676, 620006702, 620006702, 621967101,<br>621703001, 621756502, 621755902, 621755902, 621755902, 621755902, 646130268, 640463134,<br>640463134, 640463134, 640463134, 640463134, 620009566, 620009566, 620009567, 621987301,

	621967202, 621538203, 622077302, 621836501, 646130269, 640463135, 640463135, 640463135, 640463135, 640463135, 640463080, 640463080, 640463080, 620009568, 621987401, 621488601, 621538303, 621555202, 621555101, 640470011, 620002955, 620002955, 620002955, 622051701, 620007514, 620007514, 620004662, 620004148, 620004148, 620004148, 620004148, 620004148, 620006706, 620006706, 620004149, 620004149, 620004149, 620004149, 620004149, 620006707, 620006707, 640407076, 621966801, 621966801, 621994601, 621994601, 640407077, 621966901, 621966901, 621994701, 621994701, 640407080, 640407081, 640443046, 640443047, 666130005, 666130005, 666130006, 666130006, 646130037, 646130301, 646130302, 640443048, 620005201, 620005202, 620005180, 620003780, 620003781, 646120011, 622037301, 622074801, 621111803, 622327500, 620003210, 620006210, 646120012, 620006211, 621112004, 616130512, 616130513, 621946401, 621964002, 620008731, 620008731, 621113002, 616130332, 621113712, 622323800, 616130526, 616130333, 622079901, 622329200, 620003815, 622104401, 622131501, 621812201, 622045702, 620003816, 620004767, 620004750, 620006316, 621766301, 621114602, 620003817, 620004768, 620004751, 620006317, 621766401, 621115302, 620007477, 640451022, 640451023, 610411055, 610411055, 620003004, 620003004, 620003004, 620003004, 620003004, 620004133, 620004133, 620004133, 620004133, 620004133, 620004135, 620005875, 620009561, 622250001, 622419601, 622408701, 620004775, 620004776, 620008446, 620008447, 622423101, 610411056,

	620008702, 621926801, 621926801, 621116201, 621116301, 621709001, 620007519, 620009585, 620009586, 621952301, 621950101, 621967801, 621967901, 621946701, 621947901, 622044201, 620007520, 620009587, 620009588, 621952401, 621950201, 621968001, 621968101, 621946801, 621948001, 622044301, 622439001, 622419401, 620007518, 621952501, 621967701, 621931801, 640451036, 640451037, 621708501, 622078501, 621727601, 646130264, 620004152, 620007316, 620007316, 620007316, 620007316, 620008216, 620008216, 622083601, 622099301, 622130901, 622111401, 622080002, 646130265, 640444050, 640444051, 640444051, 640444071, 640444071, 640453097, 620003742, 620003742, 620003742, 620004714, 620004714, 620007317, 620007317, 620007317, 620007317, 621347901, 621116801, 621441901, 620004108, 620004108, 620007364, 620008211, 622010201, 622127601, 622172601, 620003703, 620003703, 620003703, 620004106, 620004155, 620004155, 620004707, 620005643, 620007365, 620007362, 620007363, 646130368, 646130369, 640406222, 620003003, 620003003, 620003003, 620003003, 620003003, 620004132, 620004132, 620004132, 620004132, 620004134, 620005874, 620009560, 622118402, 622118402, 622100601, 640406223, 620002977, 620002977, 620003002, 620000022, 620000022, 620001975, 620001975, 620001974, 620001974, 620004463, 620000020, 620000020, 620000021, 620000021, 621120001, 621120001, 620003495, 620003495, 620003496, 620003496, 620003554, 620003555, 620003555, 620004503, 620004503, 620004504, 620004504, 620004504, 616140099, 620002885,

	620004096, 620004096, 620005638, 621681102, 621681102, 621694602, 621693303, 621694201, 621675901, 616140101, 616140101, 616140104, 616140104, 620003921, 620003921, 620003918, 620003919, 620003919, 620003919, 620003917, 620003917, 620003917, 620003920, 620003922, 620003922, 620003916, 620003916, 620003916, 620003916, 620003923, 620003945, 620004075, 620004075, 620005425, 620005425, 620005425, 620006669, 620006669, 622079301, 621741203, 621741203, 621752801, 621752801, 616140102, 616140102, 616140105, 620003932, 620003932, 620003928, 620003926, 620003926, 620003926, 620003929, 620003929, 620003929, 620003927, 620003927, 620003927, 620003927, 620003930, 620003931, 620003931, 620003933, 620003933, 620003934, 620003935, 620003946, 620004076, 620004076, 620006670, 620006670, 620008013, 620008013, 622079401, 621742103, 621742103, 621742103, 621752901, 621752901, 620003940, 620003941, 620003943, 620003943, 620003943, 620003943, 620003943, 620003942, 620003939, 620003939, 620003939, 620003939, 620003939, 620004077, 620004077, 620004077, 620004476, 620004476, 620004974, 620005426, 620005427, 620006671, 620006671, 620008014, 621753001, 621348401, 621348401, 622295301, 622303301, 622303301, 622269501, 622274301, 622274301, 622274501, 622274501, 622353101, 622411501, 610443026, 610443026, 610443026, 622286701, 622294801, 622294801, 622290801, 622274201, 622274201, 622276801, 622296901, 622303401, 622303401, 622269601, 622275601, 622295401, 622289801, 622289801, 622274401, 622282801,

	622281501, 622270801, 622275401, 622275401, 622352901, 622368001, 610451034, 622274601, 622281601, 622270901, 610443024, 610443024, 622290701, 622303201, 622303201, 622269401, 622353001, 620009094, 622085701, 621122501, 621122601, 621122801, 616150003, 616150002, 621123301, 620006083, 620006084, 620007109, 620007109, 621124301, 616150088, 616150088, 621124603, 621124801, 621125101, 616150064, 621125401, 621125701, 616150063, 616150063, 610454083, 610454083, 621126003, 620004770, 622329300, 620005226, 620007513, 621126801, 621126501, 620004918, 616220021, 620004291, 616220017, 616220005, 616220036, 616220036, 616220006, 616220037, 616220037, 616220002, 620008287, 621134201, 621134301, 622341501, 620008358, 646220001, 616220025, 620008333, 620008649, 616220010, 610454007, 610454007, 616220011, 610454008, 610454008, 620003244, 620008620, 620008620, 620004093, 620004093, 620004093, 620004093, 620004093, 620008583, 621127501, 620007375, 620008440, 620007539, 621130301, 621130301, 621131001, 646190020, 646190018, 610462048, 610462049, 622029101, 622289101, 622289201, 622289301, 616210045, 620006826, 616210040

antiviral agent	620005140, 610444011, 610444011, 620003456, 621444601, 621444601, 621444801, 621444905, 621527604, 621527604, 621720601, 621720601, 622024801, 622024801, 610406386, 610453095, 610453095, 610453095, 610453095, 610453095, 621445001, 621445001, 610444012, 610444012, 610444112, 610444112, 610444112, 610444117, 610444117, 610453001, 610461001, 620009298, 620009298, 621142001, 621353002, 621445205, 621445301, 621445603, 621445701, 616290163, 610433006, 610444013, 610444113, 610444113, 610444113, 610453002, 610453104, 610453104, 610453104, 620003457, 620009299, 620009299, 620009300, 621353802, 621353901, 621445904, 621446001, 621446403, 621446501, 616250001, 610453009, 610453009, 610453009, 610453010, 610453010, 610453010, 610453096, 610453096, 610453096, 621633701, 610463007, 621676401, 610443081, 610443082, 620000425, 620000426, 620004975, 610412192, 610412193, 620004347, 620003516, 622054801, 622054801, 621143601, 621143601, 621143701, 621143701, 610421341, 620004998, 620006943, 621932401, 610462012, 610462012, 622292601, 622281701, 622271501, 622286101, 610443030, 622266101, 622304501, 622304501, 622286801, 622291701, 622295101, 622297701, 622287801, 622276901, 622292701, 622297001, 622301701, 622302701, 622269901, 622276101, 622304301, 622277801, 622298701, 622269301, 622287401, 622287401, 622268601, 622295901, 622293201, 622304401, 622283301, 622281801, 622271401, 622295201, 622273001, 622286501, 622275501, 622275501, 622285501, 610443041, 610443074, 610462002, 620004852,

	622083401, 622083401, 610451031, 620000454, 620000455, 620001903, 622336301, 620002413, 620002414, 620002465, 620002488, 620004355, 620005884, 622276701, 622403501, 620006802, 620007815, 620009086, 620009087, 622105001, 622105001, 622149101, 622279401, 622336201, 622363601, 622363501, 622374101, 622418801, 621144201, 621765701, 621384201, 621384201, 621657001, 621662301, 622235801, 622388001, 622408801, 622442101, 622445801, 620003679, 621144401, 620003671, 621384303, 622325900, 620006284, 620006283, 620001341, 620004633, 620004634, 620004634, 620009268, 621144901, 621384402, 621384411, 621384414, 621384424, 640461002, 620003746, 621660102, 620003761, 620003765, 620003765, 622197301, 622197401, 621972202, 621972102, 621972102, 620006495, 620006495, 620006495, 620006496, 620007574, 620008961, 620009008, 620009008, 621995901, 621146701, 621146701, 621146701, 621354901, 621354901, 621146601, 621146601, 621146502, 621146502, 660453001, 620006404, 666250002, 620000360, 620000360, 621447603, 621447501, 660453038, 660453038, 620002305, 620002305, 620002305, 620003042, 660443018, 622012101
antifungal agent	620008666, 620007031, 620004560, 620004560, 620007032, 620007032, 620007467, 620003487, 620002493, 620002494, 622375301, 640462040, 640462041, 620003489, 620002497, 622136201, 622136301

antiprotozoal agent	616410003, 616410017, 616410025, 616410025, 616410018, 621166401, 621166501, 620006817, 620007057, 620002484, 622224801, 622225701, 621167101, 622364001, 646410002

List 5. Vasopressor codes

Types of vasopressor	specific medical codes for reimbursement
vasopressin	620009273, 642410016
adrenaline	620517902, 642450005, 621371901, 620518102, 662450001
noradrenaline	620008384, 642450071

Supplementary file 2. Supplementary tables

Table S1. Background of patients, overall cohort

Overall cohort, N= 511,848	
Female gender, N (%)	207850 (40.6)
Age, median [IQR]	76.0 [66.0,84.0]
Weight, median [IQR]	52.0 [42.6,62.0]
BMI, median [IQR]	21.5 [18.7,24.6]
Elixhauser comorbidity score, median [IQR]	4.0 [0.0,7.0]
Operation during hospitalization, N (%)	109965 (21.5)
Hospital onset sepsis, N (%)	51829 (10.1)
Unit type, N (%)	
ECU	161025 (31.5)
HDU	93112 (18.2)
ICU	257711 (50.3)
Organ support on the first day of critical care unit admission, N (%)	
Vasopressors	132705 (25.9)
Mechanical ventilation	113381 (22.2)
Renal replacement therapy	32807 (6.4)

IQR = interquartile ranges, BMI = body mass index, ICU = intensive care unit, HDU = high dependency unit, EICU = emergency intensive care unit.

Table S2. Comorbidities

	18-29<br>N=8,322	30-39<br>N=11,790	40-49<br>N=23,033	50-59<br>N=38,410	60-69<br>N=86,928	70-79<br>N=142,083	80-89<br>N=157,588	90+<br>N=43,694
congestive heart failure	645 (7.8)	1141 (9.7)	2589 (11.2)	4556 (11.9)	12250 (14.1)	25068 (17.6)	36248 (23.0)	12656 (29.0)
cardiac arrhythmias	250 (3.0)	395 (3.4)	928 (4.0)	1850 (4.8)	6124 (7.0)	14373 (10.1)	19627 (12.5)	5624 (12.9)
valvular disease	251 (3.0)	376 (3.2)	732 (3.2)	1140 (3.0)	2739 (3.2)	4744 (3.3)	5726 (3.6)	1724 (3.9)
pulmonary circulation disorders	137 (1.6)	222 (1.9)	429 (1.9)	517 (1.3)	1094 (1.3)	1680 (1.2)	1608 (1.0)	379 (0.9)
peripheral vascular disorders	42 (0.5)	111 (0.9)	363 (1.6)	906 (2.4)	2768 (3.2)	5060 (3.6)	4878 (3.1)	976 (2.2)
hypertension, uncomplicated	266 (3.2)	802 (6.8)	2580 (11.2)	5684 (14.8)	14998 (17.3)	28173 (19.8)	34290 (21.8)	10499 (24.0)
hypertension, complicated	13 (0.2)	42 (0.4)	101 (0.4)	162 (0.4)	366 (0.4)	535 (0.4)	734 (0.5)	273 (0.6)
paralysis	83 (1.0)	63 (0.5)	123 (0.5)	246 (0.6)	482 (0.6)	694 (0.5)	547 (0.3)	117 (0.3)
other neurological disorders	1655 (19.9)	1536 (13.0)	2414 (10.5)	3367 (8.8)	6390 (7.4)	9717 (6.8)	9331 (5.9)	1706 (3.9)
chronic pulmonary disease	409 (4.9)	500 (4.2)	899 (3.9)	1800 (4.7)	6176 (7.1)	13364 (9.4)	14254 (9.0)	3107 (7.1)
diabetes, uncomplicated	351 (4.2)	927 (7.9)	2775 (12.0)	5737 (14.9)	14757 (17.0)	24224 (17.0)	21445 (13.6)	3986 (9.1)
diabetes, complicated	86 (1.0)	349 (3.0)	1495 (6.5)	3171 (8.3)	7217 (8.3)	9906 (7.0)	7318 (4.6)	1090 (2.5)
hypothyroidism	52 (0.6)	87 (0.7)	191 (0.8)	359 (0.9)	824 (0.9)	1395 (1.0)	1765 (1.1)	554 (1.3)
renal failure	142 (1.7)	464 (3.9)	1518 (6.6)	3481 (9.1)	9013 (10.4)	13752 (9.7)	12500 (7.9)	2645 (6.1)
liver disease	357 (4.3)	777 (6.6)	1937 (8.4)	3654 (9.5)	6349 (7.3)	7241 (5.1)	5532 (3.5)	756 (1.7)
peptic ulcer disease, excluding bleeding	229 (2.8)	354 (3.0)	661 (2.9)	1241 (3.2)	2580 (3.0)	3746 (2.6)	3440 (2.2)	822 (1.9)
AIDS/HIV	53 (0.6)	122 (1.0)	189 (0.8)	109 (0.3)	112 (0.1)	58 (0.0)	23 (0.0)	0 (0.0)
lymphoma	93 (1.1)	152 (1.3)	338 (1.5)	693 (1.8)	1930 (2.2)	2547 (1.8)	1622 (1.0)	190 (0.4)
metastatic cancer	80 (1.0)	136 (1.2)	516 (2.2)	1172 (3.1)	3141 (3.6)	4022 (2.8)	2256 (1.4)	369 (0.8)
solid tumor, without metastasis	221 (2.7)	525 (4.5)	1747 (7.6)	4512 (11.7)	13770 (15.8)	22389 (15.8)	18179 (11.5)	3215 (7.4)
rheumatoid arthritis/ collaged vascular disease	281 (3.4)	479 (4.1)	976 (4.2)	1548 (4.0)	3845 (4.4)	5702 (4.0)	3695 (2.3)	452 (1.0)
coagulopathy	683 (8.2)	1137 (9.6)	2096 (9.1)	3697 (9.6)	8462 (9.7)	12651 (8.9)	12575 (8.0)	2625 (6.0)
obesity	53 (0.6)	120 (1.0)	189 (0.8)	142 (0.4)	135 (0.2)	102 (0.1)	57 (0.0)	7 (0.0)
weight loss	20 (0.2)	38 (0.3)	103 (0.4)	189 (0.5)	361 (0.4)	459 (0.3)	482 (0.3)	146 (0.3)
fluid and electrolyte disorders	636 (7.6)	967 (8.2)	1992 (8.6)	3223 (8.4)	6649 (7.6)	10923 (7.7)	14370 (9.1)	4764 (10.9)
blood loss anemia	53 (0.6)	80 (0.7)	190 (0.8)	335 (0.9)	719 (0.8)	972 (0.7)	1011 (0.6)	221 (0.5)
deficiency anemia	133 (1.6)	269 (2.3)	559 (2.4)	782 (2.0)	1729 (2.0)	2696 (1.9)	3305 (2.1)	946 (2.2)
alcohol abuse	41 (0.5)	288 (2.4)	923 (4.0)	1564 (4.1)	1907 (2.2)	1251 (0.9)	352 (0.2)	24 (0.1)
drug abuse	16 (0.2)	29 (0.2)	57 (0.2)	40 (0.1)	28 (0.0)	19 (0.0)	3 (0.0)	0 (0.0)

psychoses	257 (3.1)	563 (4.8)	1195 (5.2)	1810 (4.7)	2802 (3.2)	2562 (1.8)	2089 (1.3)	558 (1.3)
depression	241 (2.9)	422 (3.6)	863 (3.7)	1015 (2.6)	1354 (1.6)	1964 (1.4)	1669 (1.1)	345 (0.8)

Data is presented as numbers with percentages

AIDS/HIV = acquired immunodeficiency syndrome/human immunodeficiency virus.

Table S3 Discharge destination by organ support type on the day of critical care unit admission

Age group	18-29	30-39	40-49	50-59	60-69	70-79	80-89	90+
**All patients**	**N=8,322**	**N=11,790**	**N=23,033**	**N=38,410**	**N=86,928**	**N=142,083**	**N=157,588**	**N=43,694**
Home	6254 (75.2)	8339 (70.7)	15060 (65.4)	22926 (59.7)	47459 (54.6)	68288 (48.1)	59403 (37.7)	12859 (29.4)
Facility	73 (0.9)	128 (1.1)	268 (1.2)	503 (1.3)	1623 (1.9)	4624 (3.3)	12335 (7.8)	7034 (16.1)
Hospital	1308 (15.7)	2152 (18.3)	4818 (20.9)	8857 (23.1)	21250 (24.4)	37901 (26.7)	48013 (30.5)	12942 (29.6)
Death	687 (8.3)	1171 (9.9)	2887 (12.5)	6124 (15.9)	16596 (19.1)	31270 (22.0)	37837 (24.0)	10859 (24.9)
**Vasopressor**	**N=2,236**	**N=3,757**	**N=8,452**	**N=1,5367**	**N=35,578**	**N=53,065**	**N=49,156**	**N=9,324**
Home	881 (58.2)	1540 (57.4)	3264 (52.6)	5457 (47.7)	11377 (42.6)	14237 (35.8)	9498 (25.5)	1369 (19.1)
Facility	11 (0.7)	20 (0.7)	57 (0.9)	124 (1.1)	411 (1.5)	938 (2.4)	2150 (5.8)	829 (11.6)
Hospital	301 (19.9)	542 (20.2)	1420 (22.9)	2690 (23.5)	6852 (25.6)	11255 (28.3)	12042 (32.4)	2221 (31.1)
Death	322 (21.3)	582 (21.7)	1468 (23.6)	3177 (27.8)	8093 (30.3)	13358 (33.6)	13488 (36.3)	2731 (38.2)
**Mechanical Ventilation**	**N=3,184**	**N=4,521**	**N=9,052**	**N=15,229**	**N=34,585**	**N=52,228**	**N=46,425**	**N=8,178**
Home	1239 (57.4)	1595 (52.2)	2757 (46.6)	4071 (41.8)	8507 (38.2)	10790 (31.5)	7333 (24.0)	1010 (18.5)
Facility	19 (0.9)	38 (1.2)	44 (0.7)	87 (0.9)	222 (1.0)	535 (1.6)	1119 (3.7)	456 (8.4)
Hospital	514 (23.8)	781 (25.6)	1639 (27.7)	2726 (28.0)	6345 (28.5)	10133 (29.6)	9680 (31.7)	1642 (30.1)
Death	385 (17.8)	639 (20.9)	1470 (24.9)	2852 (29.3)	7214 (32.4)	12750 (37.3)	12440 (40.7)	2349 (43.0)
**RRT**	**N=796**	**N=1,592**	**N=3,733**	**N=6,823**	**N=15,663**	**N=20,659**	**N=14,485**	**N=1,482**
Home	210 (49.6)	402 (50.2)	927 (46.9)	1491 (41.8)	2934 (36.5)	3173 (30.6)	1540 (22.2)	94 (13.6)
Facility	0 (0)	1 (0.1)	2 (0.1)	11 (0.3)	49 (0.6)	119 (1.1)	189 (2.7)	36 (5.2)
Hospital	76 (18.0)	171 (21.4)	430 (21.8)	827 (23.2)	1938 (24.1)	2738 (26.4)	2004 (28.9)	222 (32.1)
Death	137 (32.4)	226 (28.2)	617 (31.2)	1238 (34.7)	3118 (38.8)	4353 (41.9)	3195 (46.1)	339 (49.1)
**No organ support**	**N=5,267**	**N=7,018**	**N=13,095**	**N=21,105**	**N=46,958**	**N=81,759**	**N=101,305**	**N=32,760**
Home	4392 (83.4)	5580 (79.5)	9752 (74.5)	14458 (68.5)	29803 (63.5)	45758 (56.0)	43978 (43.4)	10614 (32.4)
Facility	49 (0.9)	77 (1.1)	181 (1.4)	323 (1.5)	1068 (2.3)	3308 (4.0)	9358 (9.2)	5833 (17.8)
Hospital	648 (12.3)	1072 (15.3)	2358 (18.0)	4487 (21.3)	10641 (22.7)	20712 (25.3)	29967 (29.6)	9568 (29.2)
Death	178 (3.4)	289 (4.1)	804 (6.1)	1837 (8.7)	5446 (11.6)	11981 (14.7)	18002 (17.8)	6745 (20.6)

Data is presented as numbers with percentages

RRT = renal replace therapy.
